# Feasibility and acceptability of ‘low-intensity mental health support via a telehealth-enabled network’ for adults with type 1 and type 2 diabetes: the LISTEN pilot study

**DOI:** 10.1186/s40814-023-01367-2

**Published:** 2023-07-27

**Authors:** Edith E. Holloway, Shikha Gray, Jennifer Halliday, Benjamin Harrap, Carolyn Hines, Timothy C. Skinner, Jane Speight, Christel Hendrieckx

**Affiliations:** 1 School of Psychology, Deakin University, Geelong, VIC Australia; 2 The Australian Centre for Behavioural Research in Diabetes, Diabetes Victoria, ACBRD, 570 Elizabeth Street, Melbourne, VIC 3000 Australia; 3 Institute for Health Transformation, Faculty of Health, Deakin University, Geelong, VIC Australia; 4 Deakin Rural Health, School of Medicine, Deakin University, Warrnambool, VIC Australia; 5 Diabetes Victoria, Carlton, VIC Australia; 6 La Trobe Rural Health School, La Trobe University, Flora Hill, VIC Australia; 7 Department of Psychology, University of Copenhagen, Copenhagen, Denmark

**Keywords:** Diabetes health professionals, Psychological intervention, Telehealth, Feasibility study, Brief problem-solving therapy, Diabetes distress, Mental health, Emotional support

## Abstract

**Background:**

This study examined the feasibility and acceptability of the low-intensity mental health support via telehealth*-*enabled network (LISTEN) intervention, for adults with diabetes, facilitated by diabetes health professionals (HPs).

**Methods:**

*LISTEN training*. Three HPs participated in three half-day online workshops and applied their learnings during training cases (maximum four). Competency was assessed with a validated tool and achieved ‘satisfactory’ ratings for three consecutive sessions. *LISTEN pilot.* A single-group, pre-post study (up to four LISTEN sessions) with online assessments at baseline, post-intervention, and 4-week follow-up. Eligible participants were adults with type 1 or type 2 diabetes, with diabetes distress, but excluded if they had moderate/severe depressive and/or anxiety symptoms. Feasibility was assessed via recruitment and session completion rates. Acceptability was assessed with post-intervention self-report data. Changes in diabetes distress and general emotional well-being from baseline (T1) were explored at post-intervention (T2) and at 4-week follow-up (T3).

**Results:**

Two HPs achieved competency (median training case sessions required: 7) and progressed to deliver LISTEN in the pilot study. In the pilot, *N* = 16 adults (*Med* [IQR] age: 60 [37–73] years; 13 women) with diabetes participated (median sessions per participant: 2). Twelve participants (75%) completed the post-intervention assessment (T2): 92% endorsed the number of sessions offered as ‘just right’, 75% felt comfortable talking with the HP, and 67% were satisfied with LISTEN. Perceived limitations were the structured format and narrow scope of problems addressed. Diabetes distress scores were lower post-intervention.

**Conclusions:**

This pilot demonstrates the feasibility of training HPs to deliver LISTEN, and the acceptability and potential benefits of LISTEN for adults with diabetes. The findings highlight adaptations that may enhance the delivery of, and satisfaction with, LISTEN that will be tested in a hybrid type 1 effectiveness-implementation trial.

**Supplementary Information:**

The online version contains supplementary material available at 10.1186/s40814-023-01367-2.

## Key messages regarding feasibility

- LISTEN is an evidence-based, early intervention to support the emotional well-being of adults with type 1 and type 2 diabetes, using brief problem-solving therapy (PST). Prior to this study, it was not known if the training of diabetes health professionals in LISTEN, using an online format, is feasible and the acceptability of the LISTEN telehealth program to adults with diabetes.
- Our findings demonstrate it is feasible to train diabetes health professionals to provide LISTEN and that both the online training and telehealth delivery format are acceptable. People with diabetes would like greater flexibility in the delivery of the PST steps.
- The findings highlight refinements that may enhance health professional delivery and participant satisfaction with LISTEN, including additional experiential exercises and further training/coaching in micro-counseling skills. The impact of these strategies on both intervention effectiveness and implementation outcomes will be tested in a hybrid-implementation trial.

## Background

People with diabetes are at increased risk of mental health problems, such as depression and anxiety, as well as diabetes-specific emotional issues [1–4]. The most common of these is diabetes distress, which relates to the negative emotional experiences of living with, and managing, diabetes. Diabetes distress is highly prevalent, experienced by ∼36% of the adult population with type 1 and type 2 diabetes [3], and includes feelings of frustration, worries, guilt, and/or hopelessness [5]. In Australia, of the 1.4 million people with type 1 and type 2 diabetes [6], 42% are reported to experience at least moderate levels of psychological distress [7]. Systematic reviews demonstrate that even moderate levels of distress in people with diabetes are associated with suboptimal self-care, medication taking, and glycated hemoglobin (HbA1c), leading to increased risk for macrovascular and microvascular complications [8, 9], and a 50% higher mortality rate [3, 10]. Psychological distress is both a predictor, and a consequence, of diabetes-related complications [8, 10].

Early intervention for diabetes distress and subthreshold mental health symptoms may prevent the development of more serious and intractable mental health conditions, for example persistent diabetes distress has been identified as a precursor to depression [5]. Yet, mental health care has been identified as a significant area in diabetes care needing improvement [11–13]. Around two-thirds of people with diabetes do not receive appropriate mental health support [14, 15] and fewer than 25% report that they have been offered emotional support when needed [16]. People with diabetes experience multiple barriers to receiving mental health support including stigma, cost, lack of awareness about available support [17], a lack of psychologists with an understanding of the challenges of living with diabetes [18], and a lack of (diabetes) health professionals attending to the emotional burden of long-term diabetes self-management.

Diabetes health professionals (HPs), such as diabetes educators, are well placed to deliver evidence-based strategies for the prevention and early intervention of emotional problems in people with diabetes. They understand the everyday, behavioral demands of diabetes self-management, and grasp the health and social implications of living with the condition. Adults with diabetes place value on receiving emotional support from their diabetes HPs [19, 20]. However, diabetes HPs report a lack of training, skills, confidence, time, and resources to attend to people’s emotional needs [21]. Few interventions have been designed to address diabetes distress, and there is little guidance available to inform how best to equip HPs with the important competencies.

LISTEN (low-intensity mental health support via telehealth-enabled network) was conceived as a brief, evidence-based telehealth program, facilitated by diabetes HPs. LISTEN aims to provide adults with diabetes an early intervention emotional support, centered on strengthening the person’s practical skills in problem-solving [22]. Problem-solving is an adaptive behavioral coping strategy [23] and recognized as an essential skill for effective diabetes self-management [24–26]. We adapted problem-solving therapy (PST) [22], a step-wise and structured intervention, to target problems related to living with, and managing, diabetes that contributes to emotional distress. PST has demonstrated effectiveness in improving mental health outcomes in a range of populations [27, 28]. A brief (low-intensity) version of ~4 sessions has been developed, suitable for delivery to people experiencing mild-to-moderate levels of distress and by HPs without specific mental health qualifications [29–31]. Findings from a pilot RCT in adults with diabetes and retinopathy demonstrated that brief PST reduces diabetes distress and subthreshold depressive symptoms and produces clinically significant improvements in HbA1c [32].

The aim of this study was to explore the feasibility of LISTEN, delivered via telehealth, by trained HPs and to explore its acceptability for adults with diabetes experiencing at least mild symptoms of diabetes distress in the absence of moderate-to-severe depression and/or anxiety symptoms.

## Methods

This study had two phases: (1) training diabetes HPs to deliver LISTEN and (2) a pilot study investigating the feasibility and acceptability of LISTEN. All aspects (workshops, supervision, sessions) were conducted online (e.g., via Zoom video meetings), due to the coronavirus (COVID-19) pandemic, and the physical distancing restrictions in place throughout the project.

### LISTEN training of diabetes health professionals

#### Recruitment and procedure

Three HPs volunteered to participate in the LISTEN training, including one who joined the study in case that one of the two HPs would become unavailable/withdrew). The HPs were employed at Diabetes Victoria (the Victorian Agent of the National Diabetes Services Scheme (NDSS), which is an initiative of the Australian Government, administered by Diabetes Australia). They were experienced in responding to NDSS Helpline calls for support and information for diabetes self-management. HPs were eligible to participate if they had (1) qualifications as a credentialled diabetes educator: registered nurse (RN or division 1) or accredited practicing dietitian, (2) a minimum of 12 months experience in a diabetes setting, (3) previous training or experience in supporting people with emotional problems and/or motivation to upskill to do so, and (4) capacity to undertake the training and deliver LISTEN for the study duration. HPs consented to take part in the study and completed a brief online (pre-training) survey which included questions about their professional history and reasons for participating in, and expectations of, the training. HPs also completed an online post-training survey.

#### LISTEN training program

LISTEN training comprised: (1) participation in three half-day workshops and (2) delivery under supervision to training cases.

The 3-day workshop was adapted for diabetes HPs in Australia from an established training program [22], which demonstrated high-level performance results among nurses [33–35]. A training manual and an example session narrative were provided. The online workshops comprised PST theory-informed learning modules and practical skill training for facilitating LISTEN sessions, including one opportunity to role play a session. The training also focused on strategies to strengthen HPs’ skills in showing empathy (attentive listening, reflection, summarizing) [36] and addressing common barriers to mental health support-seeking (e.g., from a general practitioner (GP) or mental health professional). The workshops were delivered by two experienced research fellows (SG and EH) with expertise and/or clinical experience in (a) psychological therapies, including PST; (b) training health professionals in mental health and emotional support strategies; (c) providing clinical supervision; and (d) the psychosocial aspects of diabetes.

Following the completion of the workshops, HPs were allocated a minimum of two, and a maximum of four, training cases. On average, HPs facilitated sessions with two training cases at a time. Training cases were adults with diabetes who met the inclusion criteria for the study (Additional file 1) and had volunteered to be a “training case”. With participants’ consent, all sessions were audio-recorded. Each session was reviewed, and structured feedback was provided to the HP during a weekly 1-h supervision session with SG.

### Pilot study

#### Study design

This was a single-group, pre-post pilot, and feasibility study with online data collection at baseline, immediately post-intervention, and 4-week follow-up (post-intervention).

#### Recruitment and eligibility

We aimed to recruit 20 adults with diabetes. Inclusion criteria were currently residing in Victoria, Australia; aged 18 to 75 years; self-reported diagnosis of type 1 or type 2 diabetes; at least mild diabetes distress (score ≥ 25 on the Problem Areas in Diabetes (PAID) scale (or a score of ≥ 2 (moderate problem) on three or more PAID items). Exclusion criteria were a score ≥ 3 on either the depression or anxiety subscales of the four-item Patient Health Questionnaire (PHQ-4), indicating moderate-to-severe depression and/or anxiety symptoms. A summary of the mental health inclusion criteria and referral pathways is presented in Additional file 1.

Prospective participants were recruited using convenience sampling through websites, e-newsletters, and social media (Twitter, Facebook) via the researchers’ affiliated professional accounts. Diabetes Victoria staff were encouraged to promote the study through similar strategies, as well as via peer support and consumer groups.

Prospective participants were directed to an online survey hosted on the Qualtrics™ platform. Those who consented to take part in the study, completed questions to determine their eligibility and, if eligible, were directed to the baseline assessment. Those who were ineligible were informed immediately using an autogenerated message and, based on their responses, were provided with links and resources to mental health support as well as an open text field for contact details if they consented to be followed up by the research team.

#### Procedure

Eligible respondents were paired with an available HP by the project coordinator (SG). The HP supported each participant over a maximum of four weekly 45–60-min sessions via phone or video call. The number of sessions was determined by the participant, based on their needs and acquisition of problem-solving skills. Sessions were offered weekly to allow participants to implement their action plan (*homework tasks*) between sessions. During each session, the HPs kept a ‘tracking sheet’ of the participant’s problem, goal, solution, and action plan and emailed a simplified version to the participant. The HPs audio-recorded at least 25% of their sessions, which were reviewed (by SG and EH) to inform feedback provided during fortnightly (and ad hoc) individual and monthly group supervision sessions. A combination of different sessions (e.g., first, second) was selected for review.

Following their final session, participants completed online post-intervention and 4-week (post-intervention) assessments.

#### Intervention

LISTEN sessions were centered on a seven-step model of PST: (1) defining the problem, (2) setting an achievable goal, (3) brainstorming solutions, (4) assessing pros and cons of solutions, (5) choosing a solution, (6) creating an action plan, and (7) evaluating outcomes. During sessions, participants received support from the HP in identifying and addressing a problem that may be contributing to diabetes distress. Participants could focus on different problems in subsequent sessions. Participants were encouraged to reframe problems that lacked a behavioral component or that were beyond their control. If participants disclosed significant distress, they were encouraged to seek further mental health support. At the end of each session, participants were prompted to create an action plan that included meaningful and enjoyable activities during the week.

### Measures and outcomes

#### LISTEN training of diabetes HPs

##### Feasibility

Recorded training-case sessions were reviewed against the Problem-Solving Treatment Adherence and Competence Scale (PST-PAC) [37] by SG and EH independently. The PST-PAC examines fidelity to technical skills, adherence to the problem-solving steps, process tasks, communication and interpersonal effectiveness (15-items), and global competence (1-item). Competency is rated from 0 (*not completed*) to 5 (*well above standard*). SG and EH discussed the PST-PAC scores until a consensus was reached. Completion of a minimum of two and maximum of four training cases, including a PST-PAC rating of at least 3 (*satisfactory*) for three consecutively rated sessions, was required for HPs to progress to delivery of LISTEN. These cutoffs were selected based on an established PST training program [22].

##### Acceptability

HPs’ satisfaction with the LISTEN training (and weekly supervision) was explored using study-specific rating scales and open-ended questions via a post-training online survey.

#### Pilot study

##### Feasibility

This is determined by participation rates, time taken to recruit *N* = 20 eligible participants (within a 4-month recruitment period), and the number of LISTEN sessions completed by participants.

##### Acceptability

Participants’ satisfaction with the intervention, and suggestions for improvement, was explored using study-specific rating scales and open-ended survey questions. Open-ended questions were adapted from previously developed items designed to explore acceptability with brief PST [30].

##### Potential psychological benefits of LISTEN

The emotional and mental health of people with diabetes was assessed at baseline, post-intervention, and at 4-week follow-up. Diabetes distress was assessed using the 20-item PAID scale [5]. Respondents rate the extent to which each issue is a problem for them on a 5-point scale (0, “not a problem”, to 4, “serious problem”). A PAID total score is calculated as the standardized sum of item scores (range 0–100) with higher scores indicating greater diabetes-specific distress. General emotional well-being was assessed with the WHO-5 Index.

Demographic and self-reported clinical data (e.g., type and duration of diabetes, treatment type) were collected at baseline only.

### Statistical analysis

Descriptive statistics were used to summarize the demographic characteristics of HPs and participants and to explore feasibility outcomes and intervention acceptability ratings. To explore potential changes (from baseline to post-intervention and 4-week follow-up) in diabetes distress (PAID) and emotional wellbeing (WHO-5), mean change scores and confidence intervals were computed. Analyses were performed using SPSS v26. Qualitative data generated from free-text responses to open-ended questions about the intervention were subjected to inductive template analysis.

## Results

### LISTEN training of diabetes HPs

#### Feasibility

Two HPs who participated in the training workshops and supervised delivery (to training cases) received a ‘satisfactory’ or higher PST-PAC rating in three consecutive LISTEN sessions within the recommended two to four training cases (median training-case sessions required to achieve competency: 7) They progressed to deliver LISTEN in the pilot study. One reserve HP did not progress to deliver the program, as further training cases were required to ensure competency, and this could not be arranged in the available timeframe.

#### Acceptability

The HPs reported satisfaction with the online training format, the training content, and duration. Areas for improvement were the inclusion of more practical exercises (e.g., role plays) and resources (e.g., video demonstration) and a greater focus on informal debriefing within supervision sessions with less emphasis on the PST-PAC scores.

### Pilot study

#### Feasibility

**Fig. 1:**
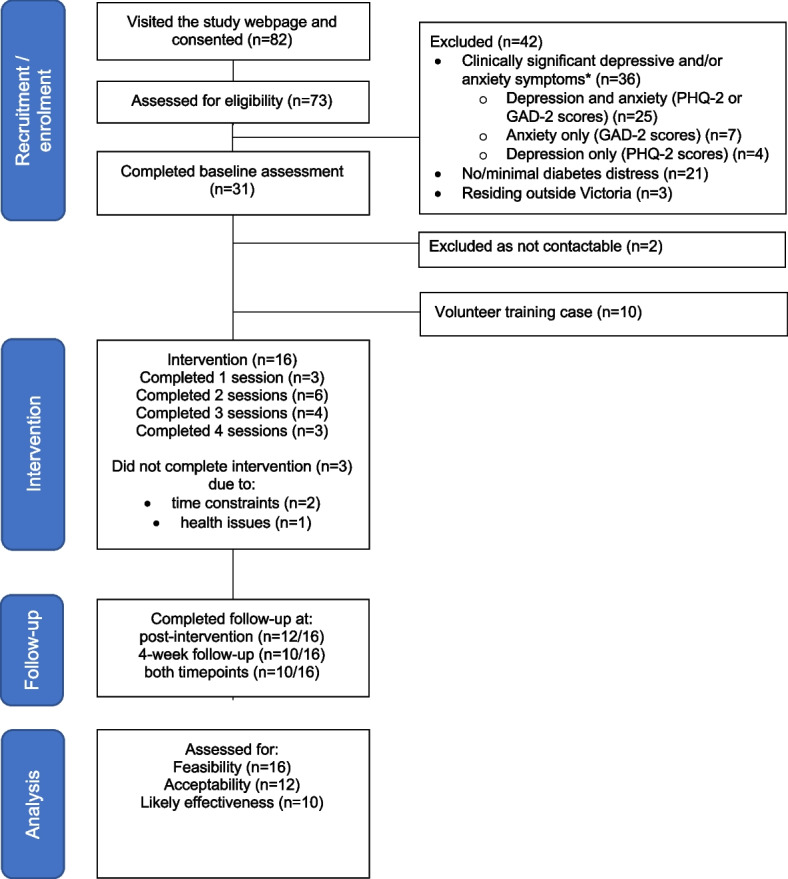
Participant flow through the study

The number of prospective participants assessed for eligibility (November 24, 2020, to May 10, 2021), enrolled, and retained at follow-up are summarized in Fig. 1. In total, 82 adults with diabetes consented to take part, nine (11%) discontinued before eligibility could be established, and 42 (58%) were ineligible (reasons are summarized in Fig. 1). Of those who were ineligible, due to moderate-severe depressive and/or anxiety symptoms, nine (21%) consented to be contacted by the project coordinator who provided resources about diabetes and emotional health and/or discussed options for accessing suitable mental health support.

Thirty-one eligible adults with diabetes were recruited into the study and completed the baseline assessment. Of these, 10 volunteered as training cases and five withdrew prior to participating in LISTEN sessions. Thus, 16 people were included in the pilot study. The median [IQR] age of participants was 60 [37–73] years, and most were women (81%). Almost two thirds (63%) had type 1 diabetes. About two thirds (62%) had had some experience (past or current) in receiving support from a mental health professional (Table 1).

**Table 1:** Demographic and clinical characteristics of adults with diabetes who participated in the pilot study (*N* = 16)

**Demographic characteristics**	***n* (%) or Median, [IQR], (range)**
Age, years (median, [IQR], range)	60 [37–73], 25–80
Sex, women (*n*, %)	13 (81.3)
Main language spoken at home, English (*n*, %)	15 (93.8)
Living situation, alone (*n*, %)	5 (31.3)
Highest level of education (*n*, %)	
School, high school or leaving certificate	2 (12.5)
Certificate/diploma/trade/apprenticeship	3 (18.8)
University or higher university degree	11 (68.8)
Employment status (n, %)	
Retired	6 (37.5)
Full-time work	5 (31.3)
Part-time work	4 (25.0)
Not working	1 (15.4)
Type of diabetes (*n*, %)	
Type 1	10 (62.5)
Type 2	6 (37.5)
Years living with diabetes (median, [IQR], range)	20 [4–26], (1–55)
Management of diabetes (n, %)	
Insulin injections	7 (44)
Insulin pump therapy	4 (25)
Diet and physical activity only	4 (25)
Oral hypoglycemic agents	3 (19)
Non-insulin injectables (e.g. GLP-1 Receptor Agonists)	1 (6)
HbA1c at last check, % (mean, range)	6.4 [6.1–8.15], (5.7–9.4)
Comorbidities (n, %)	13 (81)
Heart disease/attack	4 (25)
Neuropathy	3 (19)
Kidney damage/renal failure (including protein in urine)	2 (13)
Retinopathy	2 (13)
Vascular disease	1 (6)
Sexual dysfunction	1 (6)
Mental health conditions (n, %)	
None	9 (56)
Past diagnosis	3 (19)
Current diagnosis	3 (19)
Experience of support from a mental health professional (*n*, %)	
In the past only	6 (38)
Never	5 (31)
Currently	3 (19)
Currently and in the past	1 (6)

Missing: mental health conditions (*n* = 1) and experience of support from a mental health professional (*n* = 1)

The participants attended a median [IQR] of 2 [1–4] sessions, and the estimated average session delivery time was 60 min. Twelve (75%) returned the post-intervention assessment, and 10 (63%) returned the 4-week follow-up. Complete data for all three time points were available for 10 participants (63%).

#### Acceptability

Sixty-seven percent (8/12) of participants were satisfied with the program overall. Three in four (9/12, 75%) felt it had a positive impact on their motivation to manage their diabetes*.*

Most participants (11/12; 92%) endorsed that the maximum of four LISTEN sessions offered was ‘just right’; one preferred more sessions. The main reasons for participation in fewer than four sessions are summarized in Table 2; most (*n* = 9/12, 75%) reported that they derived as much as they wanted from < 4 sessions.

**Table 2:** Participants’ main reasons for not taking up the maximum four sessions offered^a^

Reasons given	Number of participants^b^	Illustrative quote from participants
Derived sufficient benefit from the program	4	*“I felt I had achieved what I set out to do and was confident to proceed with what I had learned”.*
Program structure and/or scope not suitable	2	*“Sessions felt very scripted and most of my issues were either unsolvable or heavily dependent on other people modifying their behaviour”.*
Work/life commitments	2	*“Work commitments meant that I could not participate in the 4th session”.*
LISTEN not suitable due to significant distress	1	*“[The health professional] ended the second session 5 min in and wished me all the best”.*^c^
Not leading to change	1	*“Was not able to properly implement changes discussed in the session”.*
Response unclear	1	-

^a^Data collected at post-intervention from *n* = 9 participants who accessed < 4 session

^b^Some participants provided more than one reason

^c^LISTEN is a low-intensity mental health intervention, thus is not suitable for severe mental health problems such as severe depression. Therefore, as per the LISTEN protocol, the health professional ended the session and referred the participant to their GP for follow-up and assessment and was provided with links to mental health services

Most participants (9/12; 75%) indicated that the duration of each session was ‘just right’, while three rated the duration as ‘too short’. Over half (7/12, 58%) preferred receiving sessions via a combination of telehealth and in-person modalities, one third (4/12) preferred phone or video, and one person preferred self-guided online modules.

Participants were generally satisfied with the support they received from the HPs; all but one (11/12, 92%) agreed (strongly or slightly) that the program was explained clearly to them and most (10/12, 83%) felt comfortable talking with the HPs about their problems associated with managing their diabetes.

Participants’ qualitative feedback suggested the usefulness of a problem-solving approach: “[LISTEN] helped me to stop and break down an issue related to managing my diabetes, rather than get overwhelmed with it” (female, type 1 diabetes). They also pointed to the positive effects of having a rapport with, and receiving empathy and encouragement from, HPs. Participants varied in their views about the structured nature of the program and the level of input from HPs as facilitators. For some, the structure was useful, but for others, it felt prescriptive, particularly when they wanted guidance/advice from the HPs. Some felt that LISTEN was limited in terms of the scope of ‘problems’ it could address, for example the unsuitability of problem-solving for persistent and/or moderate-severe emotional problems. Feedback and suggestions for adapting/improving LISTEN are summarized in Table 3.

**Table 3:** Post-intervention qualitative feedback provided by participants with diabetes (*n* = 9)

**Aspect of LISTEN**	**Participant quotes (example)**		**Recommended adaptations to LISTEN**
	**Positive aspect**	**Suggested improvement**	
Approach to solving problems	*“I liked the way the health professional was able to help me break down issues and simplify strategies and steps to work to a solution”.*	*“My problems or issues that are most concerning to me are more emotional - For example, I can’t use this problem-solving process to not be so lonely”.*	• Provide diabetes HPs with more strategies and practical application of linking emotional problems to behaviors (given that behavioral problems are amenable to PST) through vignettes, demonstrations, group reflections • Upskill HPs in referring participants on to other support (e.g., mental health professional for more complex emotional problems) • Provide further guidance to HPs on when and where to refer
Structured intervention	*“I liked the step-by-step approach. I could grasp each part before progressing to the next step”.*	*“The proposed problem-solving process did seem overly ornate, perhaps a little too inflexible and maybe a tad too much like a typical medical model”.*	• To avoid LISTEN being delivered in a ‘scripted’ and ‘formulaic’ manner, HPs to develop their own template for facilitating sessions, with cues and prompts that fit with their communication style. While the key components of the PST model need to be included for the fidelity of the intervention, the facilitation of sessions needs to feel natural and ‘conversational’ to the participant. • Ensure LISTEN is person-centered and focused on problems and goals important for the participant • Increase fluidity and flexibility with the PST steps (less prescriptive) • Focus on building therapeutic relationships (e.g., providing empathy) • Participants to set overarching goals for what they would like to get out of LISTEN (start of session 1) • Set expectations about LISTEN for the participant early on: - Revise recruitment flyer/promotional materials - Develop recruitment video (e.g., person with diabetes’ experience with LISTEN) - Develop information leaflet (i.e., what is LISTEN, participant involvement, potential benefits, testimonials
Role of health professional	*“I learnt more about myself. Someone was there to discuss, prompt and listen”.*	*“I would have liked a bit more discussion and input/ideas [from the HP] instead of just questions”.*	• Set expectations about LISTEN for the participant early on, i.e., problems, goals, and solutions should come from the participant (see above) • Fluidity and increased flexibility with the PST steps (less prescriptive)
Online format of the program	*“[It was helpful that…] I didn’t have to travel”.*	*The online format (although unavoidable these days) is impersonal.*	• Evaluate the feasibility and cost (effectiveness) of alternative modes of delivery (e.g., in-person sessions). For example, online self-paced modules + e-mail/phone support
Therapeutic relationship with HP	*“[The HP was a…] sympathetic listener… [and] was adaptable”.*	*“Maybe training for all parties involved on how to actually listen”.*	• Include additional training around micro-counseling skills, specifically focused on reflective listening and responding with empathy, to assist with rapport building and strengthening the therapeutic relationship. This needs to include the opportunity to view demonstrations and practical application of skills through role play and supervised delivery with training cases.
Not enough resources provided	*-*	*“Additional resources to help embed problem solving method, links to potential solutions”.*	• Upskill HPs in referring participants on to other support (e.g., mental health professional for more complex emotional problems or a Diabetes Educator for self-management concerns) • Provide guidance on when and where to refer

#### Potential psychological benefits of LISTEN

A reduction in diabetes distress was observed post-intervention, which continued at 4-week follow-up, with a mean difference, compared to baseline, of 16.7 (95% CI −4.3 to −29.1) and 21.5 (95% CI −8.7 to −34.3), respectively (Table 4). At post-intervention, 9/11 (82%) participants had a reduction of ≥ 5 points on their total PAID scores (average 16.8-point reduction). At the 4-week follow-up, 8/10 (80%) participants had a reduction of ≥ 5 points on their total PAID scores (average 22.0-point reduction). The PAID items with the largest decrease (0 to 4 scale) are presented in Additional file 2.

**Table 4:** Diabetes distress and general emotional wellbeing scores at baseline, post-intervention, and 4-week follow-up

**Outcome**	**Baseline (*n* = 16)**	**Post-intervention (*n* = 12)**	**4-week follow-up (*n* = 10)**
	**Mean ± SD**	**Mean ± SD**	**Mean ± SD**
Diabetes distress (PAID)	36.1 ± 14.8	19.3 ± 9.7	14.5 ± 11.5
Generic emotional well-being (WHO-5)	62.1 ± 19.6	71.3 ± 13.3	62.8 ± 26.7

*PAID* Problem Areas in Diabetes Scale, standardized sum of item scores (range 0–100) with higher scores indicating greater diabetes-specific distress; *WHO-5* The World Health Organization-Five Well-Being Index (WHO-5), total score ranging from 0 to 100, 0 representing the worst imaginable well-being and 100 representing the best imaginable well-being

There was a minimal change at post-intervention and 4-week follow-up in general emotional wellbeing compared to baseline, with a mean difference of 9.1 (95% CI 12.5–30.8) post-intervention and 0.7 (95% CI 21.6–23.0) at 4-week follow-up (Table 3).

## Discussion

This pilot is the first study to examine the feasibility, acceptability, and potential benefit of LISTEN, a brief, evidence-based telehealth intervention for adults with type 1 or type 2 diabetes experiencing diabetes distress. Our findings demonstrate that diabetes HPs can be trained to facilitate LISTEN and the online training format is acceptable. Our recruitment efforts demonstrate the potential reach and adoption of LISTEN, as well as the unmet need for support regarding the emotional and mental health aspects of living with diabetes. Participants found the content and delivery of LISTEN (by HPs via telehealth) acceptable, and two thirds reported being satisfied with the program, which was delivered to most in fewer sessions than anticipated. Preliminary evidence of benefits for reducing diabetes distress was apparent, which warrants investigation in a fully powered, randomized controlled trial.

Two HPs achieved competency within the recommended number of training cases, which corroborates previous research demonstrating successful training of non-mental health professionals in PST [30, 38]. However, we also found that each HP had varied capabilities and requirements with regard to reaching proficiency in facilitating LISTEN, thus supporting the need to allow for a flexible number of training cases for training completion. Additionally, while the use of an online format was acceptable to HPs, we found that the HPs may have benefitted from further vignettes, demonstrations, and group reflection exercises, for example to promote skills in reflective listening, providing empathy and the practical application of linking emotional problems to behaviors.

We purposely took a conservative approach to recruiting participants (i.e., primarily via social media) so as not to create demand that could not be satisfied within the parameters of a small pilot study. The recruitment period was extended due to the COVID-19 pandemic and the impact on staff scheduling. Despite this, an encouraging number of adults with diabetes visited the study webpage and consented to take part within the allocated study timeframe, suggesting there is demand, for a low-intensity HP-delivered online support service. However, a high rate of prospective participants (about half) were ineligible due to clinically significant symptoms of depression and/or anxiety (scoring ≥ 3 on the PHQ-4 depression and anxiety subscales). Refinements to the recruitment strategy may be needed to ensure that it is clear for whom LISTEN is suitable (e.g., modifying the language used in promotional materials and developing a recruitment video). Importantly, it also highlights the need for early intervention to prevent high distress and underscores the importance of having clear referral pathways to ensure that those who require higher-intensity mental health support can access support.

Post-intervention, participants reported that the content of LISTEN, the support from a diabetes HP and a telehealth format, were acceptable. This is consistent with previous findings that people with diabetes want to talk with *diabetes* health professionals about the emotional challenges they experience living with and managing diabetes (e.g., how diabetes affects their mood) [20], and they value their diabetes HPs showing empathy and acknowledging their emotional concerns [39]. It is also consistent with emerging research showing that the telehealth model of care is cost-effective, has high uptake, and has the potential to fill gaps in services and expand reach [40], While the anticipated offering of up to four sessions was deemed sufficient for learning problem-solving skills, participants recognized that more in-depth, counseling-based support would be needed for issues that are more distressing, enduring or recurring, or interpersonal in nature. Our preliminary data suggest that LISTEN may reduce diabetes distress and may have utility in supporting people to improve their capacity for coping with the daily challenges associated with diabetes, including challenges that tend to lie outside the remit of high-intensity mental health services. However, this potential effect is currently under further investigation in a randomized controlled trial.

Post-intervention qualitative feedback added further insight into the acceptability of LISTEN. Participants with diabetes valued learning PST-specific techniques, as well as the experience of having a collaborative working relationship with the HP. However, feedback also suggested an area for future consideration—how to balance the structured approach central to PST, known to be effective in other settings, with the desire for more flexible input from HPs. To enable a natural, flexible delivery of LISTEN, HPs could develop their own template for facilitating sessions with cues and prompts that fit their communication style. While the key components of the PST model need to be included to ensure the fidelity of the intervention, HPs need to develop their communication skills, and confidence in the delivery of LISTEN, to the point where their facilitation appears ‘conversational’ rather than ‘formulaic’, to avoid the impression that they are following a script. Refinements could be made to how the LISTEN program is ‘advertised’ to potential participants to ensure expectations are set early on (i.e., problems are addressed in a stepwise manner). HPs may also benefit from further training/supervision in micro counseling skills to assist in navigating the tension between delivering a manualized treatment and maintaining person-centeredness. This may include more focus during the workshops on enhancing interpersonal skills (e.g., reflective listening, empathic communication) and opportunities to apply skills during role-plays and ongoing coaching during delivery.

The study has several strengths, including the novelty of the LISTEN model enabling HPs to upskill in an evidence-based psychological intervention via a comprehensive, manualized, training program as a way of addressing known barriers to accessing mental health support. Further strengths include the use of a validated measure to ensure HPs’ fidelity to the intervention, and the provision of ongoing individual and group supervision to HPs to consolidate skills. The fact that both the facilitator training and the LISTEN sessions were delivered successfully via video meetings/telehealth is a key strength in terms of its potential to achieve reach, equity of access, and cost-effectiveness, which are particularly important in a country such as Australia, where the location of services can be a barrier to access for many. The pre-post study design allowed us to examine the feasibility of the data collection procedures and instruments, and participant tracking processes, which will be needed for the evaluation of the intervention in a fully powered two-arm hybrid type 1 effectiveness implementation trial [41].

However, this pilot study has limitations. The small sample size and the absence of a control group limit any inferences we can draw about the acceptability and potential benefits of LISTEN. Those who volunteered may not be representative of the adult population with diabetes who may benefit from LISTEN. Furthermore, the absence of longer follow-ups may increase the likelihood of bias in participant responses due to repeated measurements within a shorter time. These limitations will be addressed in a fully powered trial [41]. Furthermore, while the findings highlight key learnings to inform adaptations and improvements to the LISTEN intervention, satisfaction with the intervention and preferences for delivery need to be examined in a larger, representative sample. In the current study, the number of sessions completed by participants (range 1 to 4) was decided collaboratively between the participant and the health professional, based on the needs of the individual and the ease with which they were able to implement the problem-solving steps. Future research may also examine the minimum number of sessions needed to achieve benefits.

## Conclusions

Our findings suggest the feasibility of using an online format for training diabetes HPs in the telehealth delivery of LISTEN. Preliminary data provided favorable estimates of the program’s acceptability for both HPs and people with diabetes and the potential benefit for reducing diabetes distress. A fully powered study with a diverse sample is needed to examine (1) the suitability of both the HP training and the LISTEN intervention, (2) the (cost) effectiveness of LISTEN for reducing diabetes distress, and (3) contextual factors influencing the reach, adoption, and implementation of LISTEN delivered within existing diabetes support services.

## Supplementary Information

**Additional file 1: Table 1. **STROBE Statement—checklist of items that should be included in reports of observational studies.

**Additional file 2: Table 2.** PAID item scores at baseline, ordered by descending mean, and mean change in scores at post-intervention and at 4-week follow-up.

## Data Availability

The datasets used and/or analyzed during the current study are available from the corresponding author on reasonable request.
