# Supplementary material for: Feasibility and acceptability of ‘low-intensity mental health support via a telehealth-enabled network’ for adults with type 1 and type 2 diabetes: the LISTEN pilot study
Source: Pilot Feasibility Stud. 2023 Jul 27;9:133. doi: 10.1186/s40814-023-01367-2 (PMC10373371; doi:10.1186/s40814-023-01367-2)
Supplement: Supplementary file 2 — Additional file 2: Table 2. PAID item scores at baseline, ordered by descending mean, and mean change in scores at post-intervention and at 4-week follow-up. [file 40814_2023_1367_MOESM2_ESM.docx]

**Additional file 2.**

**Table 2.** PAID item scores at baseline, ordered by descending mean, and mean change in scores at post-intervention and at 4-week follow-up

|  |  | Mean change from Baseline | |
| --- | --- | --- | --- |
| PAID Item | Mean baseline score | Post-intervention*  **(n=11)** | 4-week  follow-up*  **(n=10)** |
| 12. Worrying about the future and the possibility of serious complications? | 2.19 | 0.61 | 1.09 |
| 20. Feeling “burned out” by the constant effort needed to manage diabetes? | 1.94 | 0.86 | 1.24 |
| 11. Feeling constantly concerned about food and eating? | 1.81 | 0.73 | 1.11 |
| 13. Feelings of guilt or anxiety when you get off track with your diabetes management? | 1.81 | 0.64 | 0.91 |
| 8. Feeling overwhelmed by your diabetes? | 1.75 | 0.83 | 1.15 |
| 17. Feeling alone with your diabetes? | 1.69 | 0.94 | 1.09 |
| 6. Feeling depressed when you think about living with diabetes? | 1.62 | 0.95 | 1.22 |
| 3. Feeling scared when you think about living with diabetes? | 1.56 | 0.64 | 0.86 |
| 9. Worrying about low blood sugar reactions? | 1.5 | 0.67 | 1 |
| 16. Feeling that diabetes is taking up too much of your mental and physical energy every day? | 1.5 | 0.42 | 0.7 |
| 19. Coping with complications of diabetes? | 1.5 | 0.5 | 0.8 |
| 7. Not knowing if your mood or feelings are related to your diabetes? | 1.44 | 0.69 | 0.84 |
| 1. Not having clear and concrete goals for your diabetes care? | 1.31 | 0.73 | 0.31 |
| 2. Feeling discouraged with your diabetes treatment plan? | 1.25 | 0.67 | 0.65 |
| 4. Uncomfortable social situations related to your diabetes care (e.g., people telling you what to eat)? | 1.25 | 0.5 | 0.75 |
| 5. Feelings of deprivation regarding food and meals? | 1.25 | 0.83 | 0.85 |
| 10. Feeling angry when you think about living with diabetes? | 1.06 | 0.48 | 0.76 |
| 14. Not “accepting” your diabetes? | 1 | 0.25 | 0.7 |
| 15. Feeling unsatisfied with your diabetes physician? | 0.75 | 0.25 | 0.55 |
| 18. Feeling that your friends and family are not supportive of your diabetes management efforts? | 0.63 | 0.3 | 0.43 |

PAID: Problem Areas in Diabetes scale: item scores range from 0 (“not a problem”) to 4 (“serious problem”); *Mean scores were lower at post-intervention and 4-week follow-up compared with baseline scores.
